# Development of 2.45 GHz Semiconductor Microwave System for Combustion Ignition Enhancement and Failure Analysis

**DOI:** 10.3390/ma15062042

**Published:** 2022-03-10

**Authors:** Yuji Ikeda

**Affiliations:** i-Lab, Inc., #213 KIBC Bldg., 5-5-2 Minatojima-Minami, Chuo, Kobe 650-0047, Japan; yuji@i-lab.net; Tel.: +81-78-958-9090

**Keywords:** microwave chemistry, microwave ignition process, microwave control

## Abstract

We developed a semiconductor microwave system to improve the ignition process in a combustion system. Under atmospheric pressure conditions, large plasma was successfully ignited by a 2.45 GHz microwave, and it is characterized in comparison with standard spark plug ignition and laser ignition. The size of the microwave power source was also effectively reduced with the minimal size (100 × 60 mm^2^) that could fit in the palm of a hand. We then prototyped a microwave plug with a diameter of 4 mm, which is smaller than the standard spark plugs for passenger cars. The design and electric field strength are discussed in detail. Combustion experiments were conducted using a motorcycle engine and an actual light vehicle, and significant fuel efficiency improvement was experimentally obtained. We investigated the wear of the plug caused by continuous operation, and efficiently improved the endurance by swinging the resonance frequency between 2.4 and 2.5 GHz. In a passenger car engine experiment using a flat panel igniter, significant fuel efficiency improvement was confirmed. Further failure analysis revealed that the ceramic was severely damaged by a large current surge.

## 1. Introduction

Ignition is the most important part of the combustion process. Optimal emission control during ignition is indispensable for reducing CO_2_ and exhaust-gas emissions [1]. We are heading towards a decarbonized society, and a wide variety of fuels are being considered [2,3,4,5]. This is a shift towards a hydrogen-based society where hydrogen transport is actively conducted [6]. Other alternatives such as ammonia combustion ideally reduce emission pollutants, but viability issues have not been resolved [7].

Aside from emission pollutants, heat generation during ignition decreases overall efficiency. Most of the ignition in a combustion system, such as spark plug and laser ignition, locally generates a high-temperature heat source. Those represented by the spark plug generate heat transfer losses through cylinder walls [8,9]. Laser ignition solves this problem by moving the flame kernel away from the combustion chamber [10,11]. However, the feasibility of laser ignition decreases in terms of the system cost.

**Figure 1:**
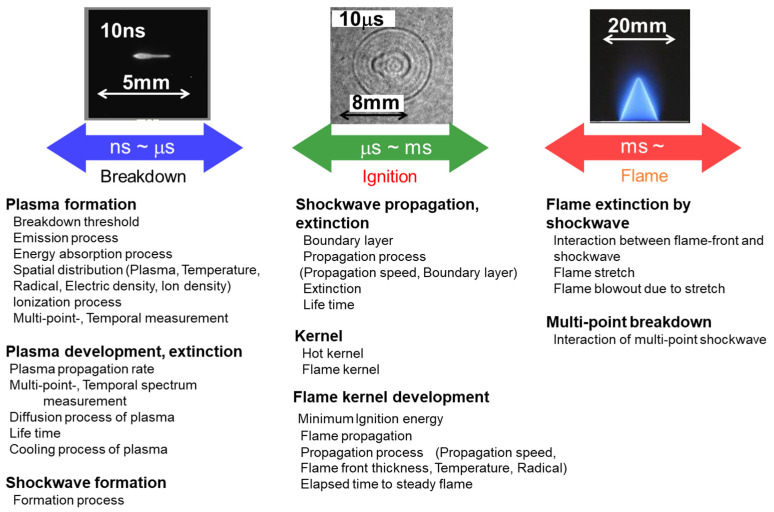
Physicochemical process of combustion.

In general, the following are important parameters for a highly efficient combustion process: stable ignition, suppression of misfire, rapid combustion, suppression of unburned fuel, miniaturization of the combustor, high output, exhaust gas heat recirculation, use of catalyst, etc. [1]. Stable ignition requires high voltage, high-energy heat source breakdown, plasma initialization, flame kernel development, and flame propagation physicochemical processes. These phenomena are summarized in Figure 1. To see the breakdown phenomenon in detail [12,13,14], it is desirable to control the energy and time similarly to laser ignition. Breakdown plasma is formed, a shock wave is generated, and a flame kernel is created. Time changes from nanoseconds to microseconds, and when it comes to the flame surface, it is a phenomenon that happens after a microsecond period. We see multipoint breakdown, ionization, and cascade.

**Figure 2:**
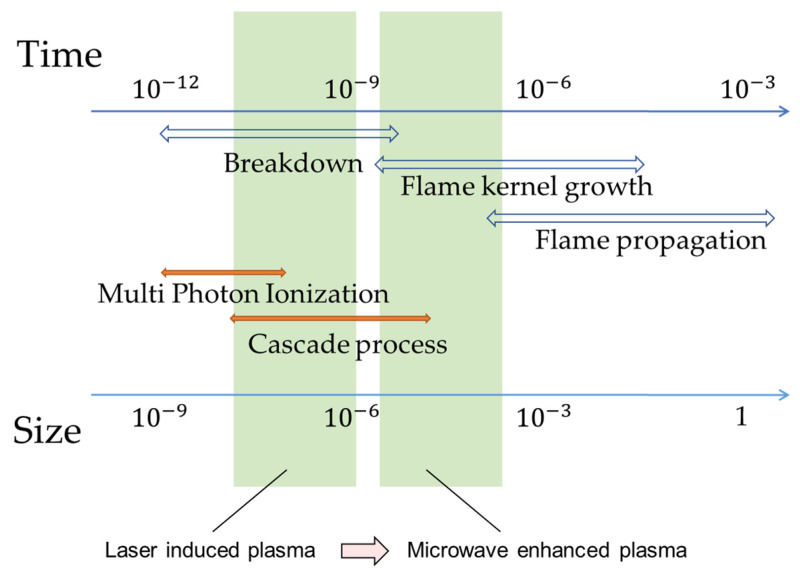
Temporal evolution of flame kernel in combustion process.

Further illustrations of laser-induced breakdown plasma, flame formation, and flame propagation are summarized in Figure 2. Here, significant enhancement and stabilization of the ignition process using microwaves are also shown. Plasma size is expanded by thousand-fold, and the plasma lifetime is prolonged by irradiating the laser-induced plasma with a microwave antenna.

**Figure 3:**
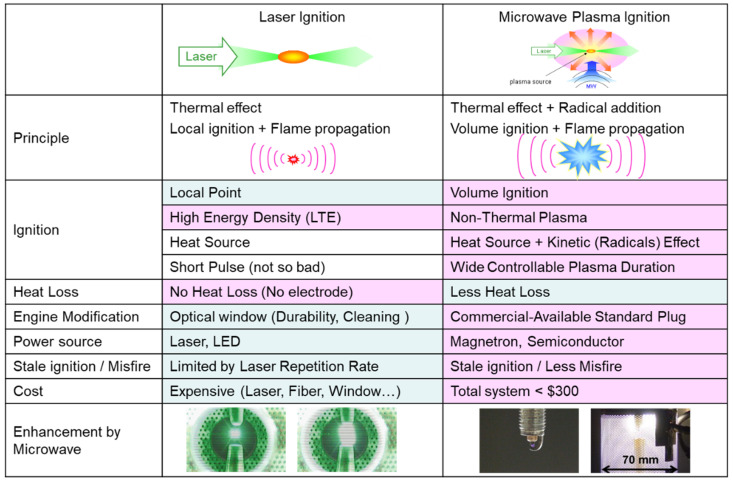
Laser ignition compared with microwave plasma ignition.

Figure 3 summarizes the characteristics of laser ignition and microwave plasma ignition using a standard spark ignition with superimposed microwave input. The advantages of each type of ignition are highlighted (pink). The volume of the kernel produced by the microwave plasma ignition is ideally larger than the laser ignition due to the addition of radicals. This type of ignition can be considered to be volume ignition instead of local-point ignition generated by laser ignition. Nonthermal plasma is produced with microwave plasma ignition, while high-density thermal plasma is generated by laser ignition. A huge advantage with using laser ignition is the nonoccurrence of the heat shrinkage problem, which is often seen in spark plugs; thus, heat loss is small. However, a window for laser light is needed, and the size and cost of the power supply are problems that are still under consideration [15]. On the other hand, a spark source coupled with a microwave system is cheaper, but the size of the entire system would be large, and integrating them into an actual engine is not yet resolved. Therefore, we are developing a compact ignition source with a novel structure that initiates the breakdown by microwaves instead of a high-voltage source, sustains the expansion of the kernel, and stabilizes the ignition by further input of microwaves [16,17,18,19,20].

This paper reports on the development of miniaturization of microwave systems, the enhancement of ignition and flame propagation, and the improvement of engine combustion efficiency. We report on the breakdown phenomenon caused by microwaves, the control of microwaves, and the temporal sustainment of breakdown plasma using semiconductor devices [21,22,23]. We developed a circuit that transmits microwaves with a coaxial cable and a breakdown plug (microwave discharge ignitor (MDI)) [24,25,26] with a resonant circuit and an antenna structure on a ceramic plate (flat panel ignitor (FPI)) [27,28,29]. We also report on the limits of combustion experiments with actual engines, countermeasures against erosion problems, and failure results.

## 2. Experimental System

### 2.1. Laser Ignition and Spark Ignition System

**Figure 4:**
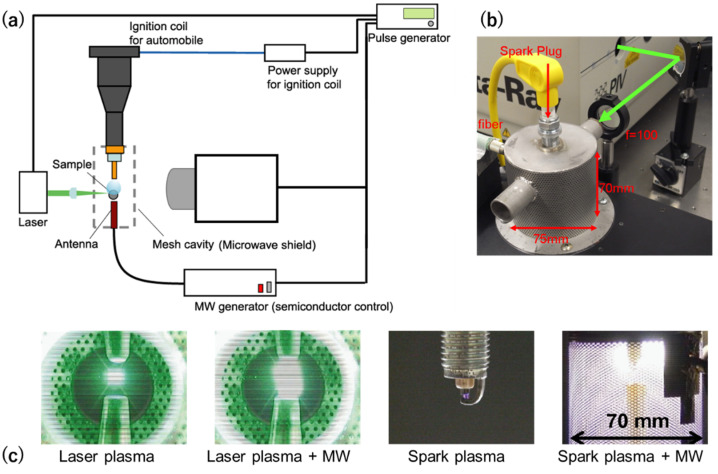
(**a**) Schematic diagram of microwave-enhanced laser ignition system; (**b**) actual image of combustion chamber of spark plug ignition plus microwave. (**c**) Enlargements of plasma plume with superimposition of microwave to laser ignition and spark ignition [22].

Figure 4a shows the schematic diagram of the microwave-enhanced laser ignition system, and Figure 4b shows the actual image of the combustion chamber of the microwave-enhanced spark ignition. For microwave-enhanced laser ignition, an Nd–YAG laser is used as a light source (Quanta-Ray ND1–004; Spectra-Physics) that is focused inside the combustion chamber to initiate breakdown. For the microwave-enhanced spark ignition, a standard spark plug was used. A coaxial antenna was used to superimpose the microwaves for both types of ignitions. The antenna was powered by a semiconductor-controlled MW generator [21,22,23]. The pulse delay generator controls the pulse input of each piece of equipment. Actual images of the plasma generated by the microwave-enhanced laser and spark ignitions are also shown in comparison with the standard ignitions without the microwave. Plasma enlargements are shown with the superimposition of microwaves for both types of ignitions. We attempted both magnetrons and semiconductor devices as microwave sources [22]. The formed plasma was significantly larger as visibly seen by the human eye (see Figure 3). Plume size varied depending on the emission spectrum. This photo was taken without a wavelength filter.

### 2.2. Semiconductor Microwave Generator

**Figure 5:**
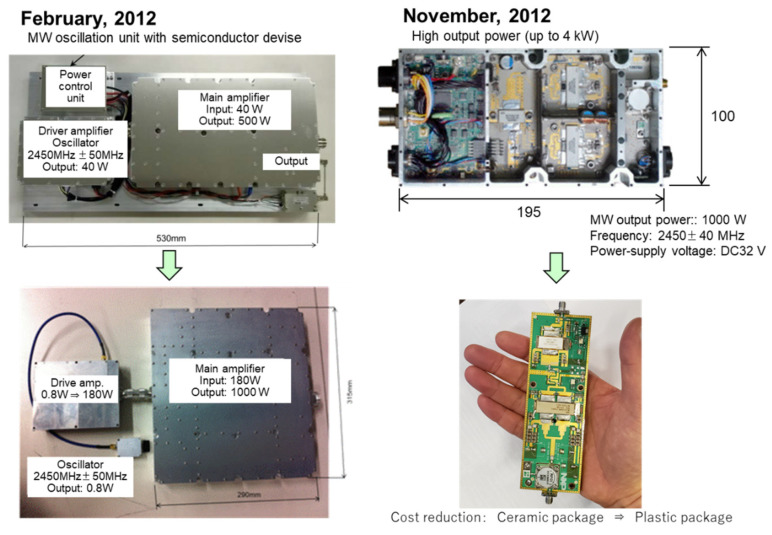
Size reduction of microwave oscillator.

Oscillation in semiconductor devices rather than magnetrons is essential for energy efficiency and reducing heat generation. Figure 5 shows the development in the reduction in semiconductor size. We developed this microwave oscillation system in 10 months with the latest model (100 × 195 mm^2^), achieving less than half the size of the previous model (530 × 230 mm^2^). The original model includes a power control unit, a driver amplifier oscillator, and the main amplifier generating a maximal output of 500 W. The latest model has a standard output of 1000 W, and a simple combination of 4 oscillators can generate a total of 4 kW. A simple arrangement of the circuitry downsized the product to a size of 60 × 100 mm^2^. This version has the size of the palm of a typical hand and fits inside the hood of a passenger car when mounted on an actual device. In the future, further improvements can reduce the production cost by using plastic packages instead of ceramic packages.

### 2.3. Antenna Characteristics

**Figure 6:**
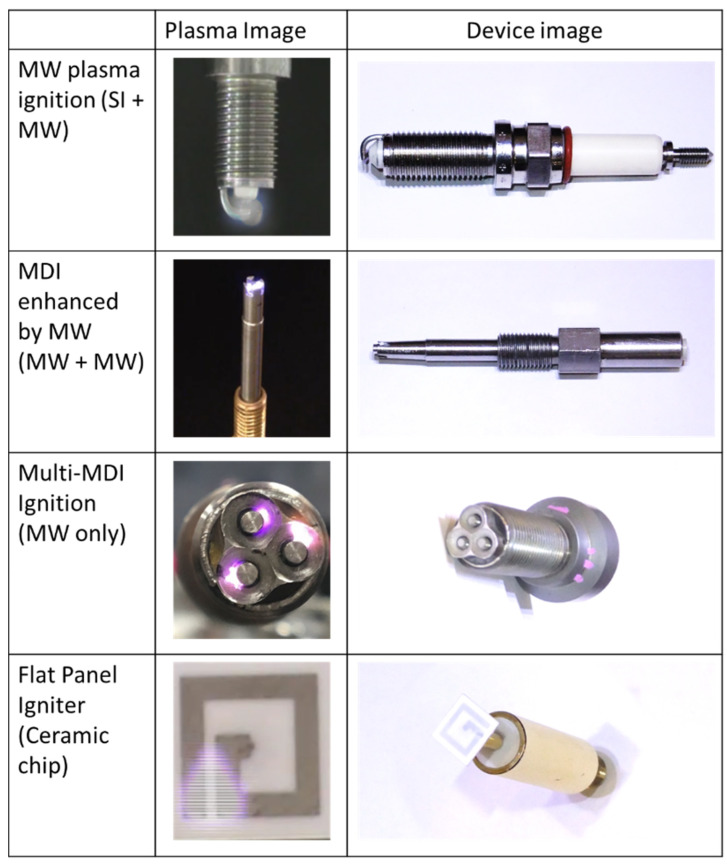
Comparison of spark plug, microwave discharge igniter (MDI), and flat panel igniter (FPI). There are multiple variations of the ignition process spark plug and MDI depending on the energy input.

Figure 6 shows the comparison of the antenna that we developed for the superimposition of MW compared with a standard spark plug. The standard spark plug had small plasma generation, but it was the simplest and cheapest system. A modified spark plug sustained by an integrated microwave antenna (MW plasma ignitions) produce a significantly larger plasma compared with the standard spark plug, but it also requires a more expensive system due to the need for two power sources: (a) high voltage source for spark plug breakdown and (b) microwave source for sustaining the enlarged plasma. This led to the development of two major types of antennas that only require one microwave source for the breakdown and sustainment of the plasma: the microwave discharge igniter (MDI) and the flat panel igniter (FPI). The MDI transmits high frequencies from a microwave oscillator into a ceramic oscillator that concentrates the MW transmission in the central electrode where the plasma breakdown is initiated [28]. The FPI, on the other hand, uses a ceramic chip antenna where the antenna structure is printed in a ceramic plate. Plasma breakdown is initiated between the smallest distanced antenna prints. The single-point MDI and FPI compete with the standard igniter in terms of system simplicity and costs. Both types of ignitions have the advantage of creating larger plasma. Two variations of the MDI using triple igniters (3 × MDI and M12) only use one oscillation pattern for breakdown and sustaining the plasma. The 3 × MDI variation uses three separate MDI antennas that are placed linearly. The M12 antenna integrates the 3 MDI into one structure. Significant improvements in the plasma size are observed for both types of antennas. The multipoint ignition of gas fuel is also superior in terms of ignition characteristics and stability.

In this paper, we conducted an experiment using an M12 plug fitting the whole system in the hood of a passenger car. The cost, ignition control, and maintenance were comparable to those of standard spark plugs with a standard lifetime of about 100,000 km. The balance of effectiveness, function, material, and cost made the development challenging. We considered using the system not only for gasoline-powered vehicles, but also for diesel engines and gas engines.

### 2.4. MDI Detailed Design and Erosion

**Figure 7:**
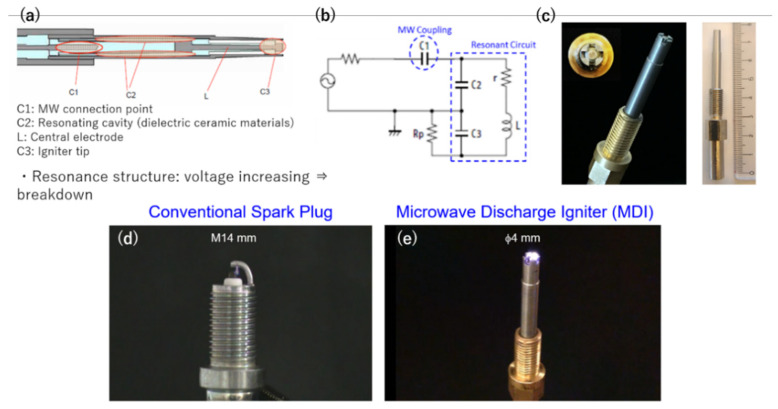
(**a**) Schematic diagram, (**b**) equivalent circuit, (**c**) and actual image of microwave discharge igniter (MDI). Actual scale size is compared with that of a (**d**) conventional spark plug. (**e**) Actual ignition of MDI.

Regarding the development of MDI, we developed one that is smaller than the smallest spark plug diameter used in gasoline-powered vehicles. This is because the degree of freedom in designing the engine cylinder can be improved. Another advantage of using a smaller diameter spark plug is the increase in distance of the plasma from the engine cylinder that can lessen the heat loss. Figure 7a–c show the schematic diagram, equivalent circuit, and actual images of the MDI. Two concentric metal rods and a ceramic resonator formed the MDI. Breakdown occurs in the concentric space at the tip of the antenna, and pulsed microwaves are continuously sent to sustain the plasma temporally and spatially. The equivalent circuit shows how the capacitive values of the MW connection point and the resonant cavity affect the central electrode. With the optimum MW coupling and resonant capacitance, the ignition is observed as shown in Figure 7e. This produced plasma was larger, denser, and more intense compared with the spark produced in the standard spark plug in Figure 7d.

**Figure 8:**
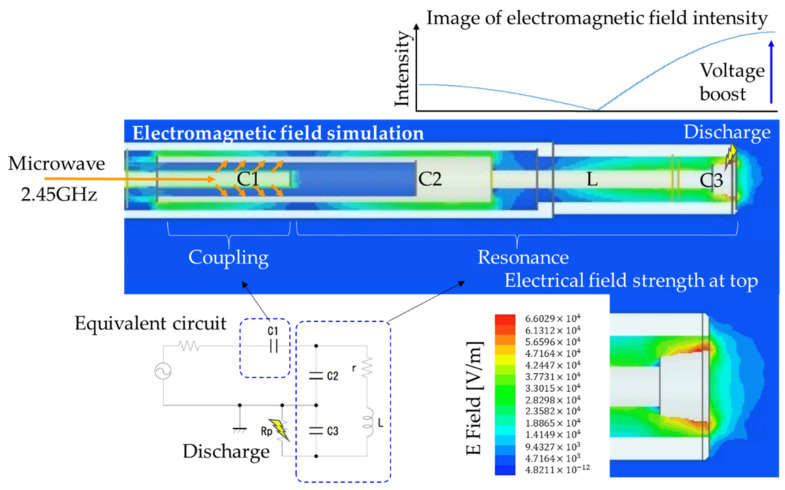
Electric-field strength simulation inside microwave discharge igniter (MDI). The capacitance of the microwave coupling point C1 was varied for maximum E-field in ignition tip C3. The resonant cavity C3 and inductance L comprised the resonant circuit.

To further understand the ignition process in the MDI, electric-field analysis using HFSS is shown in Figure 8. The microwave was transmitted from the MW oscillator into the C1 structure. The MW creates a localized electric field in the whole MDI structure through proper coupling in structure C1. The C1 structure was optimized for generally low distribution of the electric field in the resonant structure C2 and L, and maximum electric field strength concentration in the discharge tip, C3. The high electric field strength at the tip causes the breakdown of plasma. The combination of C2 and C1 is the most difficult parameter tuned. The performance of MDI increases with a larger C2 diameter (data not shown). However, dimensions C2 and C3 were determined by the overall MDI diameter of 4 mm. The issue of misfire was not observed with this type of igniter. Impedance matching was not performed in MDI.

**Figure 9:**
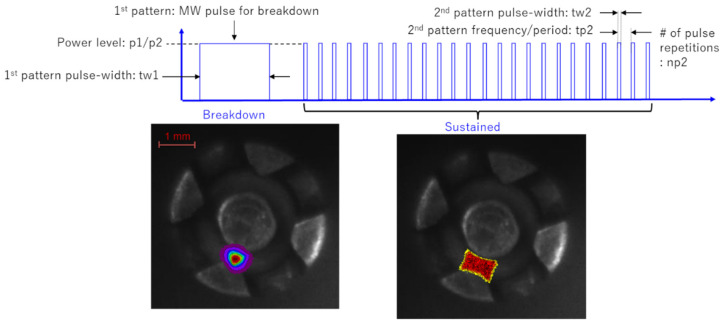
Oscillation pattern of microwave input of microwave discharge igniter (MDI) initiates plasma breakdown and sustains ignition for a long period [30].

When injecting microwaves in a pulsed manner, a few microseconds of energy are applied for breakdown, which creates a pilot fire, shown on the lower left corner of Figure 9. When a pulsed microwave is injected there, the plasma expands, as shown in the photo on the right, moves between the electrodes, and is sustained.

It was necessary to produce multiple microwave pulses instead of a signal microwave pulse to optimize the duty ratio. Each pulse was set to 0.1 mJ or less, and pulse width was controlled to be 50–100 ns. The required energy for the breakdown and that to sustain plasma is very different. It takes a certain amount of time to form a flame after the breakdown. For that purpose, it was found that sustaining the plasma for a longer period is more efficient than creating a high-energy heat source. If the pulse interval is too short, the plasma cannot be sustained. If it is too long, the temperature of the plasma rises, resulting in unnecessary energy consumption and the material melting of the spark plug itself. If the ignition energy used for two different engines is compared to that of the existing spark plug, the total fuel consumption is improved. Therefore, as little energy as possible is required. It is necessary to sustain the plasma with short pulses of microwaves. 

## 3. Results

### 3.1. MW on Spark Plug

**Figure 10:**
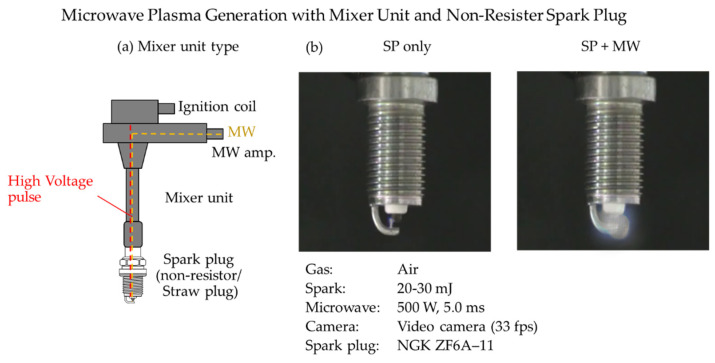
(**a**) Schematic diagram of microwave-assisted spark plug. (**b**) Actual images of plasma generated by spark plug with and without microwave input.

Two types of actual engines were used: 125 cc of gasoline for motorcycles, and 660 cc for light vehicles [29,31]. The used spark plug with microwave, shown in Figure 10, indicated the integration of the MW antenna into a high voltage spark plug for the breakdown of plasma. This system is a mixer unit type, in contrast to a system where the external microwave is added into a separate MW antenna. Spark energy was 20–30 mJ, and the microwave gave an energy of 500 W peak for 5.0 ms. The plasma emission in the atmosphere is expanded by microwaves. Using this system, we first conducted an experiment to expand the lean limit of a motorcycle engine. The coefficient of variation (COV) is reduced by about 5% to improve fuel efficiency and suppress the expansion of engine combustion fluctuations.

**Figure 11:**
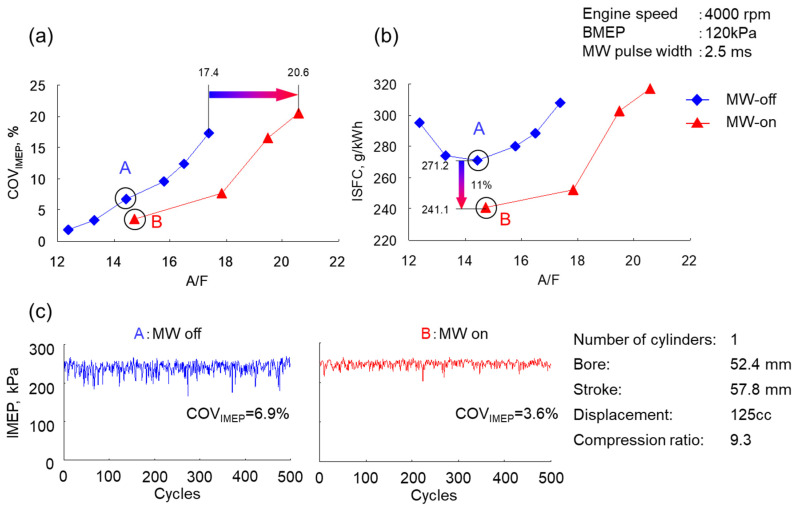
Effects of microwave to (**a**) coefficient of variation (COV) and (**b**) indicated specific fuel consumption (ISFC) in the spark plug. (**c**) Indicated mean effective pressure (IMEP) is the same with and without the MW input.

The engine speed of the gasoline engine was 4000 rpm, and the indicated mean effective pressure (IMEP) had a low load of 120 kPa. Results of constant microwave superposition of 2.5 ms for varied air/fuel (A/F) ratio are shown in Figure 11. In normal operation, the A/F is about 14.5, and there is stable operation that is stoichiometric. By inserting microwaves, this was great, and the dilution limit was expanded. Indicated specific fuel consumption (ISFC) improved by 11% in terms of fuel economy. These are the ones in which the flame propagation is accelerated, and the fuel consumption is improved by stable ignition and flame expansion.

Most motorcycle engines are air-cooled, and the temperature of the spark plug attached to the engine is higher than that of a water-cooled passenger car engine. An 11% improvement in indicated specific fuel consumption (ISFC) was obtained under this experimental condition, but it was not the same under other engine rotation conditions. The higher the engine speed was, the shorter the ignition period and the shorter the flame propagation time were. Furthermore, intake efficiency decreased, and the amount of air used for combustion decreased, so fuel efficiency tended to decrease.

### 3.2. Single vs. Multipoint MDI

**Figure 12:**
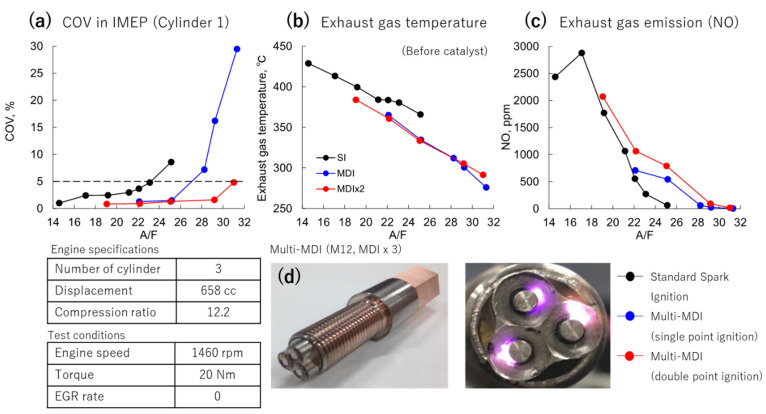
Comparison of (**a**) coefficient of variation (COV), (**b**) exhaust gas temperature, and (**c**) NO production in standard spark plugs, single-point microwave discharge igniter (MDI), and double point MDI. (**d**) Actual images of used multi-MDI.

Next, using a spark plug that integrates two sets of this MDI system with a gasoline engine for a light vehicle, we conducted an experiment to expand the combustion dilution limit. This engine had three cylinders, and a pressure sensor was inserted into each cylinder to measure combustion efficiency. Figure 12 shows the volatility COV of the engine output: IMEP obtained from the pressure of the first cylinder of the three cylinders. Combustion efficiency was examined with a COV value of 5% as a threshold. For a standard spark plug, the limit was an A/F ratio value of about 23. However, when one set of MDI was activated in the spark plug, this value was expanded to 28. Furthermore, when two sets of MDI were activated, it was greatly expanded to 31. This is an immense improvement in fuel efficiency. Combustion did not become unstable. The exhaust gas temperature of the engine also decreased. Furthermore, the NO value in the exhaust gas was greatly improved. This engine uses a three-way catalyst, but the fact that the NO value dropped to almost a single value is a remarkable achievement.

### 3.3. Endurance Test of MDI

**Figure 13:**
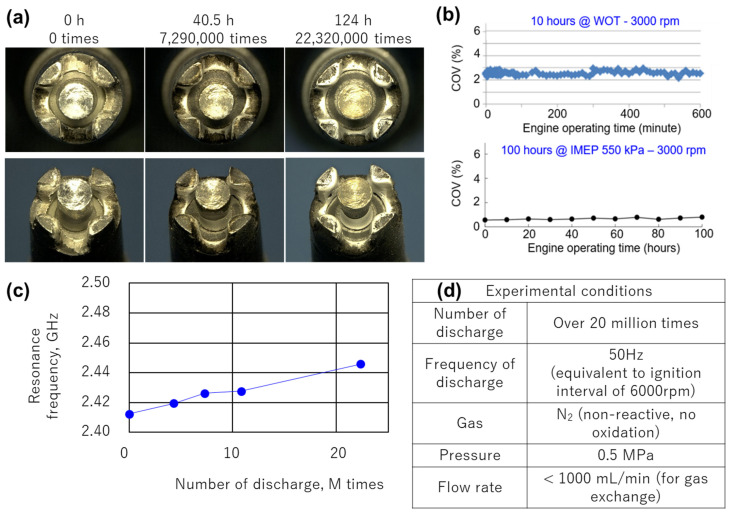
(**a**) Actual images of microwave discharge igniter (MDI) under wide throttle opening (WOT) conditions at 3000 rpm for 0 h (0 ignitions), 40.5 h (7.29 ignitions), and 124 h (22 million ignitions). (**b**) Effects of engine operation time on coefficient of variation (COV) were insignificant. (**c**) Effects of number of discharges to resonance frequency for (**d**) these experimental conditions.

Figure 13 shows the endurance test result of this MDI. The photo shows a new product under wide throttle opening (WOT) conditions at 3000 rpm for 40 and 124 h, and 124 h means 22 million ignitions. The volatility COV of indicated mean effective pressure (IMEP) when the output was operated at 550 kPa for 100 h was 1% or less, which was excellent. Even under WOT conditions, fluctuation was about 2%, and there was no problem in practical operations. However, material erosion occurred. The distance between electrodes became narrow. Commercially available spark plugs can be operated for 100,000 km, and only 0.1 mm or less is allowed because, when the gap between electrodes becomes wide, discharge energy and its probability change, and stable ignition cannot be achieved. Wear due to electric discharge also occurs in MDI. When the gap is changed, the resonance wavelength changes. The resonance wavelength is shown in the figure. When the number of ignitions was taken on the horizontal axis and the resonance wavelength was measured, it was 2.41–2.45 GHz. Since the used microwave oscillator could freely control the transmission frequency up to 2.40–2.50 GHz, this problem of gap expansion due to wear can be solved, and the life of MDI cleared that of commercial passenger cars.

### 3.4. FPI Combustion Promotion Effect and Component Destruction

**Figure 14:**
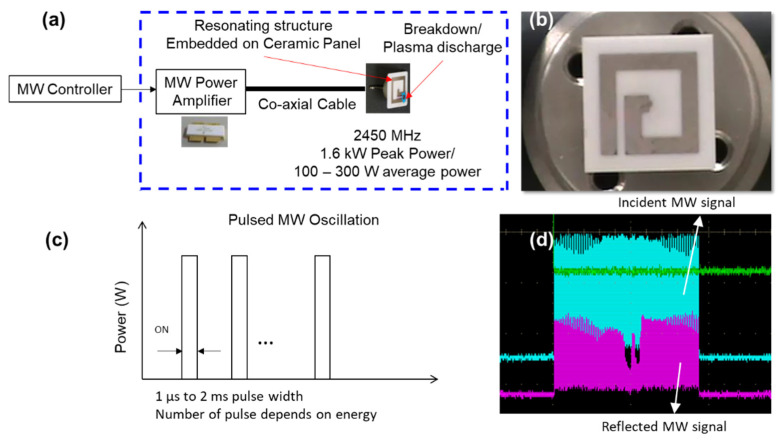
Flat panel igniter (FPI) (**a**) schematic operation, (**b**) actual image, (**c**) schematic oscillation pattern, (**d**) actual oscillation pattern.

Figure 14 shows the actual image of the FPI and the schematic diagram of the implementation of the antenna. Microwave power was transmitted using a coaxial cable from the microwave power supply to the antenna. An input of MW pulsed oscillation of 1.6 kW peak to peak was performed which generated a breakdown and plasma generation at the antenna and sustainment of nonequilibrium plasma. When the forward wave and the reflected wave were measured, about 2/3 input power contributed to the plasma generation.

**Figure 15:**
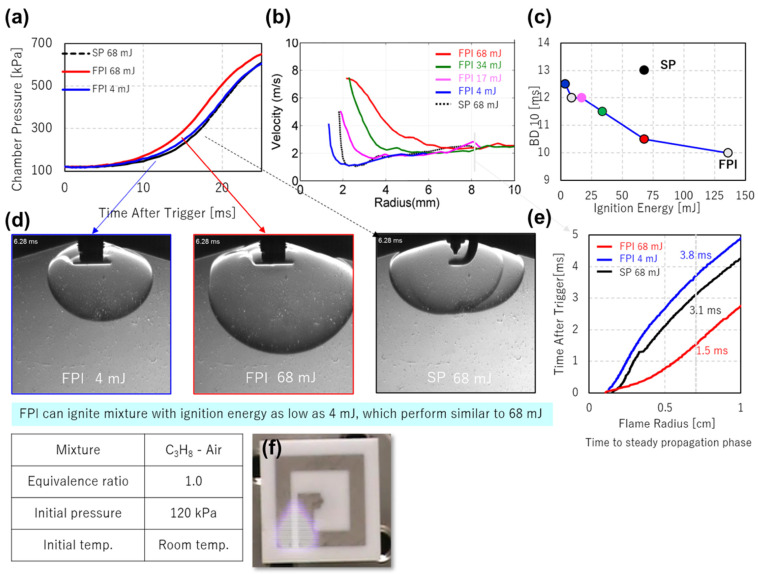
Effects of (**a**) time after trigger on chamber pressure, (**b**) flame kernel radius on plume velocity, (**c**) BD10 (pressure increase of 10%) in flat panel igniter (FPI). (**d**) Actual images of flame kernel after 6.28 ms for the low energy (4 mJ) FPI, and high energy (68 mJ) FPI and spark plug (SP). (**e**) Correlation of flame radius vs. time after trigger shows a shorter time (1.5 ms) to enlarge the flame kernel in FPI. (**f**) Top view image of flame kernel [31].

Figure 15 shows the experimental results of using the FPI in a constant combustion chamber [28] using propane. The flame propagation in the chamber was measured under atmospheric pressure and standard conditions. A comparative experiment was also conducted at 68 mJ using a standard spark plug. FPI energy results using the same input energy of 68 mJ and minimal ignition energy of 4.0 mJ are also shown. Compared to spark plugs, FPI 68 mJ forms flame nuclei faster and pressure rises earlier.

The magnitude change of the flame spread from 68 to 4.0 mJ is indicated by the radius of the flame kernel. In parameter BD10 (pressure increase of 10%) used for the flame spread of combustion, the larger the input energy was, the faster the flame spread. The initial flame propagation velocity at the minimal energy of 4 mJ was as small as 2 m/s. Compared to conventional spark plugs, this FPI has a relatively large degree of freedom in size, and shape can greatly contribute to the degree of freedom in designing cylinders, pistons, and plug positions.

### 3.5. Failure Analysis

**Figure 16:**
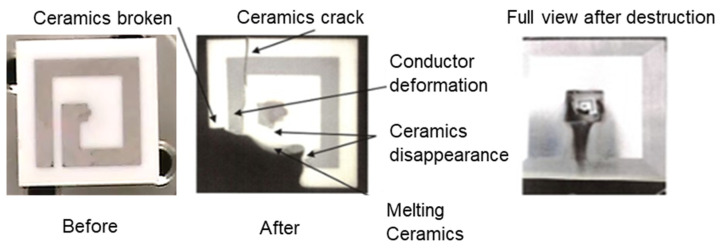
Failure analysis shows ceramic melting after 4 A current surge. Normal operations only require 0.3 A.

An FPI destruction experiment was conducted. At the same level of input energy as a standard spark plug, ignition probability was 100%, there was no problem, and it can be used for life. Therefore, a large current was applied to try to destroy it. As a result of changing the current from 0.3 to 4.0 A in normal operation, it was destroyed, as shown in Figure 16. It glowed red and the ceramic was destroyed. Furthermore, cracks were generated in the remaining ceramic part. The energy was concentrated by the large current and was input to the plasma, and the temperature became high. This failure analysis is indispensable for applications to actual device development because it is vital to take measures against malfunction of the oscillation circuit due to individual differences, noise, overcurrent in an accident, lightning, etc. The thickness of the ceramic plate was 0.8 mm, but it should be increased to several millimeters, the antenna material should be tungsten, the reflected wave of microwaves should be monitored, and the safety circuit should be installed. 

## 4. Conclusions

A microwave semiconductor system was successfully developed to improve the ignition process in a combustion system. Large plasma was confirmed compared to a spark plug under atmospheric pressure conditions. A smaller microwave ignitor of 4 mm in diameter was developed, and a microwave generator was also developed in hand-carrying size. Practical experiments were demonstrated both in practical passenger car engines of a motorcycle engine 125 cc and small passenger gasoline engine 660 cc. Remarkable lean limit extensions were observed in both engines.

Erosion problems were tested in the engine and could be controlled by changing the resonant microwave frequency of 2.4 to 2.5 GHz. A ceramic plate antenna was designed and it demonstrated its excellent combustion ignition performance. Further failure analysis revealed that the ceramic itself was severely damaged by a large current when it broke.

## Data Availability

Not applicable.
